# Strongest untreated mycelium materials produced by *Schizophyllum commune* dikaryons

**DOI:** 10.1007/s11274-025-04582-6

**Published:** 2025-10-23

**Authors:** Antonio d’Errico, Jeroen G. van den Brandhof, Anna Bogomolova, Han A. B. Wösten

**Affiliations:** Microbiology, Department of Biology, Utrecht University, Padualaan 8, Utrecht, 3584 CH The Netherlands

**Keywords:** Basidiomycete, Dikaryon, Fungus, Mycelium material, *Schizophyllum commune*, Young’s modulus

## Abstract

Monokaryons of *Schizophyllum commune* are preferred to produce mycelium materials due to their inability to form fruiting bodies and thereby producing uniform materials. However, a single gene mutation in such monokaryons can negatively impact biomass, chemical composition, and mechanical properties of the mycelium material. Dikaryons contain two instead of a single nucleus per hyphal compartment, thereby promoting higher phenotypic stability in the case of recessive mutations. Here, biomass formation and mycelium material properties of four *S. commune* dikaryons were compared to a monokaryon commonly used in mycelium material production. To this end, mycelium was grown in three different growth media. The monokaryon yielded a biomass of 3.11–9.31 g L^−1^, an ultimate tensile strength (*σ*) of 4.62–7.77 MPa, a Young’s modulus (*E*) of 0.38–0.64 GPa, and an elongation at break (*ε*) of 1.51–1.93%. Most dikaryons exhibited a strikingly higher *σ* (19.55–47.29 MPa), a similar or higher *E* (0.69–2.06 GPa) and *ε* (2.09–7.92%), and a lower or similar biomass (1.48–7.71 g L^−1^). Notably, *σ* values up to 47.29 and 47.16 MPa were obtained for the diakaryotic *S. commune* strains 139 and 351, respectively, making them the strongest untreated mycelium materials reported so far. Results suggest that this high strength is caused, at least in part, by a high amount of KOH-soluble cell wall components and by low sc3 expression.

**Supplementary Information:**

The online version contains supplementary material available at 10.1007/s11274-025-04582-6.

## Introduction

Mycelium-derived materials have emerged as a class of biobased, biodegradable materials with potentially a broad range of applications. For instance, pure mycelium materials could be used as a substitute for leather or textile (Ivanova 2022). Pure mycelium material is obtained by growing a fungus on a solid substrate or in static or dynamic liquid media (Appels et al. 2018; 2020; d’Errico et al. 2024; Wijayarathna et al. 2022; van den Brandhof et al. 2025). To this end, low quality waste streams from agriculture, horticulture, or forestry are used. Alternatively, fungi can be grown on defined media with simple carbon and nitrogen sources such as monomeric sugars and ammonium. After growth on a solid substrate, a mycelium skin is harvested from the air-substrate interface. A mycelium sheet can also be isolated from the liquid-air interface in the case of a liquid static culture, while a mycelium pulp is obtained after separating the mycelium from the medium in case of a liquid shaken culture (Appels et al. 2018; 2020; d’Errico et al. 2024; Wijayarathna et al. 2022; van den Brandhof et al. 2025). This pulp can be casted and dried, which also results in mycelium sheets. Alternatively, the mycelium pulp is processed into yarns, which can be used to produce woven textile-like materials (Svensson et al. 2021;2024).

Changing growth parameters or deleting genes can change the material’s properties such as the density (*ρ*), hygroscopicity, elongation at break (*ε*), ultimate tensile strength (*σ*) and the Young’s modulus (*E*) (Antinori et al. 2020; Appels et al. 2018; Haneef et al. 2017). For instance, deletion of the *sc3* hydrophobin gene of *Schizophyllum commune* results in materials with properties similar to polymers, while the wild-type mycelium displays properties similar to natural materials (Appels et al. 2018).

The basidiomycete *S. commune* (Kleijburg and Wösten 2025) has been used to study pure mycelium materials (Appels et al. 2018; 2020; d’Errico et al. 2024; [Bibr CR7][Bibr CR10]; Kleijburg et al. 2023; van den Brandhof et al. 2024). It has a short life cycle (from spore to spore) of about a week, which can be completed on defined media, even in 96-wells plates. Moreover, it can be genetically modified (see e.g. Vonk et al. 2019) and a high quality genome sequence is available (Marian et al. 2022; Ohm et al. 2010). Its cell wall is also well-characterized, which consists of an inner rigid core of α-(1,3)‐glucan, β‐(1,3)(1,6)‐glucan, fucan, branched mannose, and β‐(1,4)‐chitin and an outer mobile layer of β‐(1,3)(1,6)‐glucan, mannan, α‐glucan and protein (Ehren et al. 2020; Safeer et al. 2023).

The monokaryotic *S. commune* strain 4–39 is routinely used in pure mycelium material production (Appels et al. 2018; 2020; d’Errico et al. 2024; [Bibr CR7][Bibr CR10]; van den Brandhof et al. 2024). The main reason for this is that monokaryons do not produce (early stages of) fruiting bodies under any condition, thereby providing a homogeneous material surface. However, the presence of a single haploid nucleus per compartment make monokaryons like strain 4–39 sensitive for genetic mutations (Raper and Miles 1958; d’Errico et al. 2025c). This may impact biomass production and the properties of the mycelium material. By contrast, the presence of two haploid nuclei per hyphal compartment in dikaryons is expected to promote phenotypic stability (Clark and Anderson 2004; Kües 2000). Fertile dikaryons of *S. commune* are formed when two *S. commune* monokaryons fuse that have different *matA* and *matB* mating type loci (Wösten and Wessels 2006). These nuclei do not fuse in the vegetative mycelium, this only happens in the basidia, but rather each hyphal compartment contains one nucleus of each parent. Such dikaryons display a “monokaryotic” phenotype when grown in liquid shaken cultures (Schuurs et al. 1998) or when grown in static cultures in the dark and under high CO_2_ (Wösten and Wessels 2006). By contrast, fruiting bodies are formed in static cultures when they are grown in the light and under atmospheric CO_2_.

Here, biomass formation and mycelium material properties were assessed of four dikaryotic strains of *S. commune* that were grown in three different media using the monokaryotic strain 4–39 as a reference. Dikaryons produced similar or lower biomass and materials with a similar or higher *ε* when compared to the monokaryotic strain, while *ρ*, *E* and *σ* of the dikaryotic mycelium materials were generally higher. In fact, sheets produced from the dikaryotic strains 139 and 351 displayed the highest *σ* values of untreated mycelium materials reported so far.

## Materials and methods

### Strains and culture conditions

The *S. commune* 4–39 monokaryon (*MAT*A41*MAT*B41, CBS 341.81) and the H4-8 (*MATA*43*MATB*41, FGSC no. 9210) (Ohm et al. 2010a b), 139, 176, and 351 dikaryons (CBS 152882, CBS 152883, and CBS 152884, respectively) were used in this study. The latter three strains resulted from a screen for biomass production of dikaryotic strains (Kleijburg 2025). Pieces of mycelium from the periphery of 7-day-old colonies were transferred from a −80 °C stock to 50 mL Greiner tubes containing 20 mL medium. To this end, *S. commune* Minimal Medium (SCMM) (Dons et al. 1979), Ammonium Minimal Medium (MM-N) (Kleijburg et al. 2023) and Production Medium (PM) (van Wetter et al. 2000) were used. SCMM contains per liter 22 g glucose monohydrate, 1.32 g (NH_4_)2SO_4_, 0.5 g MgSO_4_·7H2O, 0.12 mg thiamin, 1 g K_2_HPO_4_, 0.46 g KH_2_PO_4_, 5 mg FeCl_3_·6H_2_O, and trace elements according to Whitaker. Asparagine that is used in SCMM is replaced for 0.01 M (NH_4_)_2_SO_4_ in MM-N and PM, while PM also contains a 4-fold higher concentration of KH_2_PO_4_ and K_2_HPO_4_ when compared to SCMM. After cultivation in the dark at 30 °C and 50 rpm (Eppendorf Innova S44i, Thermo Fisher Scientific, MA, USA; https://www.thermofisher.com) for 5 days, cultures were macerated for 30 s at 18,000 rpm in a total of 100 mL of the respective medium using a Waring Blender (Waring Laboratory, Torrington, England, www.waringlab.com*).* The macerated mycelium was incubated for 24 h in a 250 mL Erlenmeyer flask at 30 °C and 200 rpm (Eppendorf Innova S44i, Thermo Fisher Scientific). This pre-culture was macerated at 18,000 rpm for 30 s. A 2 mL aliquot of the pre-culture was centrifuged for 10 min at 10,000 g and the pellet was used to determine the wet weight of the mycelium. A 1 g wet weight mycelium aliquot was used to inoculate 2 L Erlenmeyer flasks containing 1 L of the respective medium. Cultures were grown for 7 days in the dark at 30 °C and 200 rpm. A total of 15 different conditions were tested (Table 1) and all cultivations were conducted in triplicate.

**Table 1:** The different mycelium materials that were made in this study by using different strains and media. The different materials are named according to the medium that was used (S, M, and P) followed by the strain name

	Medium		
Strain	SCMM (S)	MM-N (M)	PM (P)
4–39	S4-39	M4-39	P4-39
139	S139	M139	P139
176	S176	M176	P176
351	S351	M351	P351
H4-8	SH4-8	MH4-8	PH4-8

## Production of mycelium sheets

The mycelium of 7-day-old 1 L cultures (about 15–90 g wet weight L^−1^ and corresponding to 1.48–9.31 g dry weight L^−1^ (Table 2)) was filtered using 200 mesh nylon cheesecloth (HobbySter, www.bol.com), washed with 3 volumes demineralized water (dH_2_O), resuspended in 1 L dH_2_O, and blended for 6 s with an immersion blender (Kenwood kMix Triblade Hand Blender, www.kenwoodworld.com). The resulting suspension was filtered through Miracloth^®^ (Merck Millipore, www.merckmillipore.com) in a Büchner funnel with a diameter of 90 mm (SH4-8 and M4-39) or 120 mm (all other materials) that was connected to a vacuum pump (Leybold, Divac 1.2 L, Cologne, Germany) that applied a vacuum of 93 mBar. The harvested mycelium films were dried at room temperature (RT) on a flat surface between semi-permeable cellophane (Embalru, www.embalru.nl). Once dry, the mycelium films were separated from the cellophane.

**Table 2:** Biomass, thickness, density (*ρ*), ultimate tensile strength (*σ*), young’s modulus (*E*), and elongation at break (*ε*) of mycelium sheets of 7-day-old cultures. The different samples are named according to the medium (S, M, and P) followed by the strain name. In addition, each material is indicated with a letter a to o. The letters in superscript in each column refer to these materials and show which other materials are statistically different from a given material. For instance, the thickness of material S139 (indicated by b) is only different to material P4-39 (indicated by k), while the thickness of material S4-39 (indicated by a) is not different from any other material. The values are presented as mean ± sem. Superscript letters indicate statistically significant differences with samples with different letters in the same column (*p* ≤ 0.05)

Sample	Biomass (g L^−1^)	Thickness (mm)	ρ (kg m^−3^)	σ (MPa)	E (GPa)	ε (%)
S4-39 (a)	9.31 ± 0.28^b, d−j, m^	1.12 ± 0,04	672 ± 39^c−e, g−j, l−n^	7.77 ± 0.91^b−e, g,I, j,l, n^	0.61 ± 0.05^b, c,g, i,j, l,n^	1.89 ± 0.30^b, e,n^
S139 (b)	4.96 ± 0.32^a, c,g, j−l, n,o^	0.17 ± 0.00^k^	1289 ± 112^a, f,k, o^	47.29 ± 5.40^a, c,d, f,h−m, o^	1.73 ± 0.25^a, f,k, o^	6.51 ± 1.30^a, c,d, f−m, o^
S176 (c)	7.65 ± 0.89^b, e−h, j^	0.30 ± 0.01^k^	1170 ± 84^a, f,k, o^	25.90 ± 4.31^a, b,f, k,n, o^	1.69 ± 0.29^a, f,k, o^	2.45 ± 0.49^b, e^
S351 (d)	5.76 ± 0.27^a, f,g, j^	0.39 ± 0.00^k^	1110 ± 55^a, f,k, o^	20.75 ± 2.03^a, b,e−g, k,n^	1.52 ± 0.34^fk^	2.60 ± 0.43^b, e^
SH4-8 (e)	3.46 ± 0.83^a, c,k, l,n, o^	0.36 ± 0.02	1314 ± 105^a, f,k, o^	34.80 ± 3.46^a, d,f, h,k, m,o^	1.42 ± 0.13^k^	7.92 ± 1.16^a, c,d, f−o^
M4-39 (f)	3.11 ± 0.70^a, c,d, k,l, n,o^	0.71 ± 0.01	690 ± 87^b−e, j,l−n^	6.38 ± 0.89^b−e, g−j, l−n^	0.47 ± 0.16^b, c,d, g,I, j,l, n^	1.51 ± 0.25^b, e,n^
M139 (g)	2.18 ± 0.21^a−d, i,k−o^	0.22 ± 0.01^k^	1274 ± 106^a, f,k, o^	36.40 ± 6.15^a, d,f, h,k, m,o^	2.06 ± 0.36^a, f,k, m,o^	3.05 ± 0.53^b, e^
M176 (h)	3.77 ± 0.32^a, c,k, l,n, o^	0.26 ± 0.00^k^	1169 ± 43^a, f,k, o^	20.01 ± 2.81^a, b,e−g, k,n^	1.13 ± 0.18	2.09 ± 0.40^b, e,n^
M351 (i)	5.83 ± 0.27^a, g,j^	0.24 ± 0.01^k^	1140 ± 58^a, f,k, o^	29.10 ± 3.04^a, b,f, k,n, o^	1.66 ± 0.19^a, f,k, o^	2.21 ± 0.30^b, e,n^
MH4-8 (j)	1.48 ± 0.04^a−d, i,k−o^	0.21 ± 0.01^k^	1177 ± 73^a, f,k, o^	26.81 ± 2.96^a, b,f, k,n, o^	1.69 ± 0.12^a, f,k, o^	2.31 ± 0.20^b, e,n^
P4-39 (k)	7.77 ± 0.80^b, e−h, j^	1.29 ± 0.02^b−d, g−j, l−n^	514 ± 99^b−e, g−j, l−n^	4.62 ± 0.50^b−e, g−j, l−n^	0.38 ± 0.04^b−e, g,I, j,l, n^	1.93 ± 0.40^b, e,n^
P139 (l)	7.60 ± 0.16^b, e−h, j^	0.30 ± 0.00^k^	1266 ± 58^a, f,k, o^	32.39 ± 5.12^a, b,f, k,n, o^	1.58 ± 0.14^a, f,k^	3.10 ± 0.62^b, e^
P176 (m)	5.49 ± 0.34^a, g,j^	0.25 ± 0.01^k^	1233 ± 66^a, f,k, o^	19.55 ± 3.30^a, b,e−g, k,n^	1.05 ± 0.26^g^	3.29 ± 0.93^b, e^
P351 (n)	7.61 ± 0.35^b, e−h, j^	0.22 ± 0.01^k^	1356 ± 105^a, f,k, o^	47.16 ± 3.50^a, c,d, f,h−m, o^	1.67 ± 0.50^a, f,k, o^	4.78 ± 0.67^a, e,f, h−k, o^
PH4-8 (o)	7.71 ± 0.34^b, e−h, j^	0.63 ± 0.00	720 ± 59^b−e, g−j, l−n^	12.14 ± 2.05^b, c,e, g,I, j,l, n^	0.69 ± 0.13^b, c,g, i,j, n^	2.12 ± 0.20^b, e,n^

### Material properties

Biomass of sheets or dog-bone shape samples was determined using an Entris^®^ II analytical balance (Sartorius, www.sartorius.com). A minimum of 3 dog-bone shaped samples were cut from the central part of each sheet of mycelium. Thickness of each dog-bone shaped sample was measured at 3 random positions using a Heidenhain MT1281 electronic calliper (Heidenhain, www.heidenhain.com). Tensile tests were conducted using three technical replicates of three biological replicates per condition. Tests were performed at RT using a LS5 Universal testing machine (AMETEK Sensors LS5, Berwyn, USA, www.ametek.com) equipped with a 1 kN load cell. Samples were subjected to a preload force of 0.5 N and stretched at a speed of 10 mm min^−1^. Datapoints of stress-strain curves were continuously recorded with a total of 500 measurements. *σ* (MPa) was obtained by dividing the maximum force (N) by the area of the cross-section (mm^2^), *ε* (%) was calculated as the percentage of the difference of the gauge length before and after the test using the sudden sample failure (expressed as a drop from maximal stress to ≈ 0 MPa over a strain interval of ≈ 0.05%) as the abortion criterium, *E* (GPa) was determined in the linear section of the stress/strain curves (*E* values were only taken below 0.25% strain; ASTM International, 2017), while *ρ* (kg m^−3^) was calculated by dividing the mass of each specimen by its volume.

### Material composition analysis

Pieces of mycelium sheets were freeze-dried and ground to a powder using a tissue homogeniser (TissueLyser II, Qiagen, Germany, https://www.qiagen.com/). To separate the water- and KOH-insoluble cell wall fractions, powdered materials were sequentially treated with water and KOH (Ehren et al. 2020; Kleijburg et al. 2023) (Supplemental Fig. 1). To this end, 200 mg mycelium powder was washed four times with 40 mL dH_2_O, each wash followed with a 10-min centrifugation at 10,000 g (Kleijburg et al. 2023). This water-washed cell wall fraction was freeze-dried and weighed using an Entris^®^ II analytical balance. Subsequently, the water-washed cell wall fraction was extracted two times with 40 mL 1 M KOH at 60 °C for 20 min to obtain a KOH-soluble and KOH-insoluble cell wall fraction. The KOH-insoluble cell wall fraction was rinsed twice with dH_2_O. Subsequently, the sample was washed for 12 h in dH_2_O at 4 °C. Following this step, the KOH-insoluble fraction was freeze-dried and weighed (see above). Experiments were performed using biological triplicates.

### Quantitative real-time PCR

Biological triplicates of mycelium from 3-day-old SCMM cultures were ground with liquid nitrogen using a pre-cooled mortar and pestle. RNA was isolated by mixing 100 mg powdered mycelium with 1 ml TRIzol (Thermo Fisher Scientific). After incubating at room temperature for 5 min, the sample was mixed with 200 µl chloroform and incubated for 3 min at room temperature. Samples were centrifuged at 12,000 g for 15 min at 4 °C and the RNA in the aqueous phase was precipitated by mixing with 1 ml isopropanol and incubating for 10 min at 4 °C. This was followed by centrifugation (12,000 g, 15 min, 4 °C), after which the pellet was washed with 1 ml 75% ethanol, centrifuged (12,000 g, 5 min, 4 °C), air-dried for 5 min at 65 °C, and resuspended in 100 µl RNase-free water. RNA was purified by adding one volume of 4 M LiCl, followed by incubation overnight at 4 °C. Precipitated RNA was collected by centrifugation (12,000 g, 20 min, 4 °C), washed with 1 ml 75% ethanol, centrifuged (12,000 g, 5 min, 4 °C), air-dried (5 min, 65 °C), and resuspended in 25 µl RNase-free water at 65 °C for 10 min. RNA concentration was determined using a NanoDrop 1000 Spectrophotometer (Thermo Fisher Scientific) and cDNA was synthesized using 200 ng total RNA and the RevertAid RT Reverse Transcription Kit (Thermo Fisher Scientific) according to the manufacturer’s instructions. The resulting cDNA was diluted 1:10 prior to use in Quantitative real-time PCR (qPCR). qPCR was performed in a Hard-Shell^®^ 384-Well PCR Plate (Bio-Rad, CA, USA; www.biorad.com) with two technical replicates in a total volume of 5 µl, using the Power SYBR™ Green PCR Master Mix (Thermo Fisher Scientific) on a CFX Opus 384 Real-Time PCR system (Bio-Rad, CA, USA). The qPCR consisted of a single step at 95 °C for 10 min, followed by 40 cycles of 95 °C for 15 s, 59 °C for 15 s, and 60 °C for 30 s. Melting curve analysis (55–95 °C at 0.5 °C) was performed to verify amplicon specificity. Primer pairs CTCTCCCCTCACTGTCATC/TTGATCAGCCCGTTGAATTG and GAGGCACTACTTCCAGAACAC/GCTCGTCCTCGTTAAGCA were used for qPCR of *sc3* and *arf1*, respectively, and were spanning exon-exon junctions, yielding amplicons of 90 bp (*sc3*) and 113 bp (*arf1*). Primer efficiency was assessed in duplicate using serial dilutions (1:10 to 1:1000) of cDNA from the 4–39 monokaryon. The primer pairs had efficiencies of 92.4% (*arf1*) and 83.9% (*sc3*). Cycle threshold (Ct) values were determined using the default settings in CFX Maestro Software (Bio-Rad). Ct values of *sc3* were normalized to the reference gene *arf1* (d’Errico et al., 2025c) and calculated for relative change compared to the 4–39 monokaryon using the Pfaffl model (Pfaffl, 2001).

### Statistical analysis

Statistical analysis was carried out using IBM SPSS Statistics version 29.0.1.0 (171) (IBM Corporation, New York, USA). Data were subjected to one-way ANOVA, followed by a Tukey’s post hoc test (*p* ≤ 0.05). Levene’s Test confirmed homogeneity of variances across groups (*p* > 0.05). Shapiro-Wilk tests indicated no significant deviations from normality (*p* > 0.05). Linear regression was used to assess the relationship between the measured parameters (*p* ≤ 0.05).

## Results

### Biomass and material properties of S. commune strains

The monokaryotic strain 4–39 was grown on SCMM, MM-N and PM, resulting in mycelium dubbed S4-39, M4-39 and P4-39, respectively (Table 1). The dikaryotic strains H4-8, 139, 176, and 351 were grown in the same three media, resulting in mycelium that was coined in the same way; the name of the strain preceded by the code of the medium (S, M, or P). Highest biomass was obtained for S4-39, which amounted 9.31 g L^−1^ after 7 days of growth (Table 2). Biomass of M4-39 was lower than that of S4-39, while biomass of P4-39 was not statistically different from that of S4-39. Biomass of P139, P351, PH4-8, and S176 was also not different from S4-39. All the other conditions produced significantly lower biomass, with MM-N resulting in the lowest biomass amounts.

**Fig. 1:**
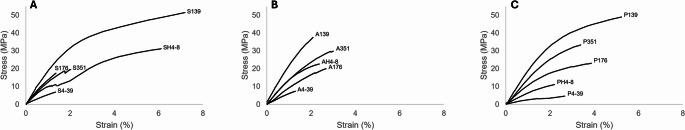
Representative stress-strain curves of the materials produced from *S. commune* strains in SCMM (A), MM-N (B), and PM (C) (see Table 1). Sample fracture always coincided with ultimate tensile strength

Materials produced from the control S4-39 displayed a *ρ* of 672 kg m^−3^, a *σ* of 7.77 MPa, an *E* of 0.61 GPa and an *ε* of 1.89% (Table 2). The properties of 4–39 were not different when the strain was grown in MM-N or PM. *ρ* of all dikaryon materials except for PH4-8 was significantly higher than that of 4–39, ranging from 1140 kg m^−3^ (M351) to as high as 1356 kg m^−3^ (P351). Similarly, all dikaryons except PH4-8 produced significantly stronger materials compared to 4–39, with a 2.5- to 10-fold higher *σ*. The highest *σ* was recorded for S139 and P351, with 47.29 MPa and 47.16 MPa, respectively. Also, most dikaryon materials had a higher *E* when compared to 4–39 with values ranging from 1.58 GPa (P139) to 2.06 GPa (M139) (Table 2). Only *E* of S351, M176, P176, and PH4-8 were not different from that of S4-39, while SH4-8 only differed from P4-39. The *ε* values of the dikaryon materials were generally not different from those of 4–39. Only the S139, P351, and SH4-8 materials had a higher *ε* than that of 4–39 with values ranging between 4.78% and 7.92%. Stress-strain curves of 4–39, 139, and 351 show the effect of medium and strain on both strength and ductility of the materials (Fig. 1). Together, the medium had generally a low effect on mechanical properties of the different strains. Data suggest that PM has the lowest performance, especially compared to SCMM.

**Fig. 2:**
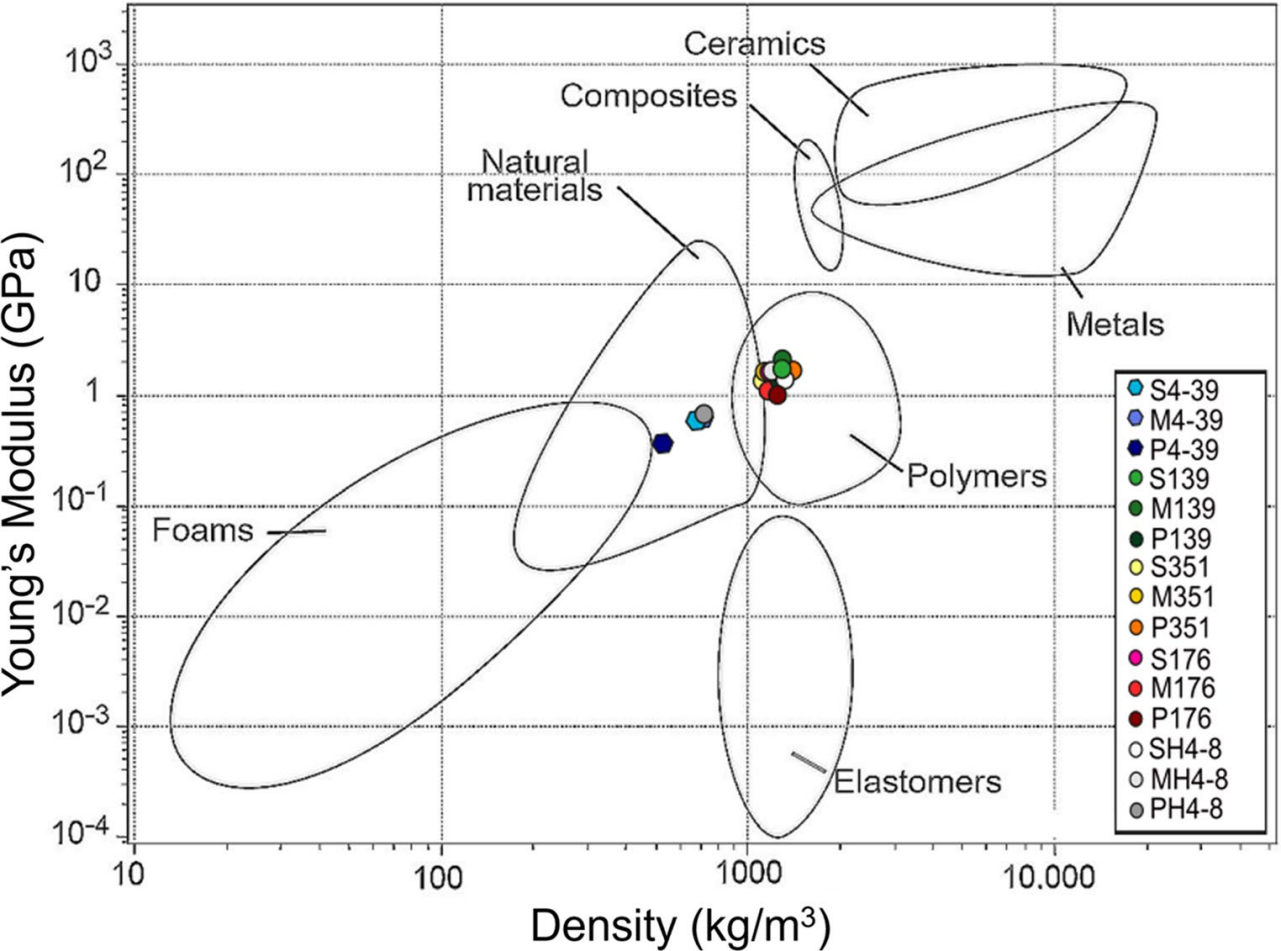
Classification of the materials derived from monokaryons (hexagons) and dikaryons (circles) in a material property chart depicting Young’s modulus and density. Adapted from Ashby (2005).

Simple linear regression analysis was used to find predictors of the mechanical properties of the materials. A strong positive relationship was found between *ρ* and *σ* (R² = 0.65), while *ρ* and *ε* showed a weak positive relationship (R² = 0.23). Plotting *E* and *ρ* revealed two types of materials in the Ashby chart (Fig. 2). A group of weaker materials consisting of PH4-8 and the three 4–39 materials (M4-39, P4-39, S4-39) was classified within the natural materials, resembling wood. All the other materials were grouped in the class of polymers with properties similar to for instance polycarbonate (PC), polyethylene terephthalate (PET), and nylon.

## Material composition as a predictor of mechanical properties

**Fig. 3:**
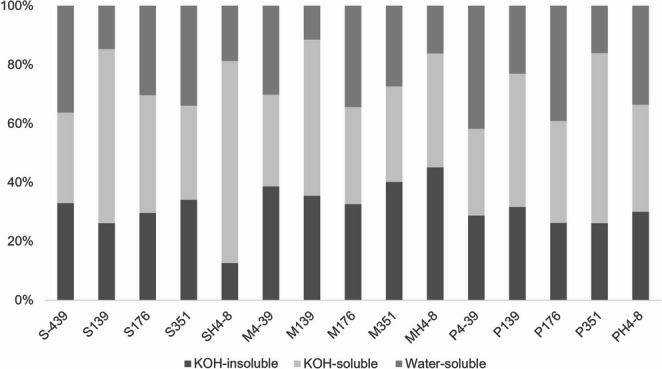
Composition of mycelium materials derived from *S. commune* strains. Water-washed cell walls comprise the KOH-soluble and KOH-insoluble material fraction. KOH-extracted cell walls correspond to the KOH-insoluble fraction

**Fig. 4:**
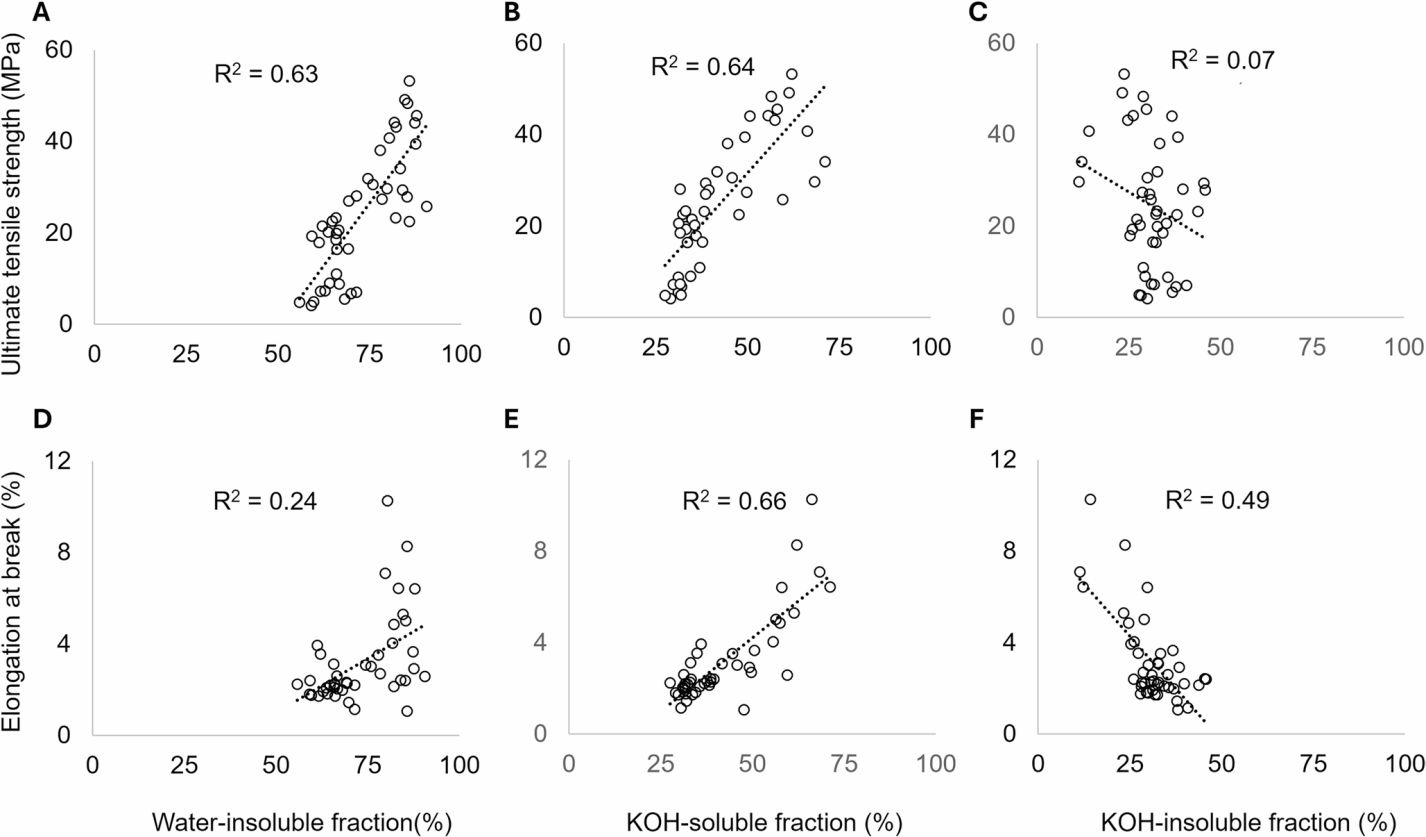
Ultimate tensile strength and elongation at break as functions of the percentage of the water-insoluble (i.e. water-extracted cell walls) (A, D), KOH-soluble (B, E), and KOH-insoluble (i.e. KOH-extracted cell walls) (C, F) fraction of the cell walls

Macerated mycelium was washed with water, after which the remaining biomass was measured. Based on this, two groups of materials were distinguished. One group, consisting of S139, SH4-8, P139, P351, M139, and MH4-8, displayed a content of water-insoluble components ranging between 77.0% (P139) and 88.5% (M139) (Fig. 3, Supplementary Table 1). Materials in the second group, however, consisted of 58.2% (P4-39) to 72.6% (M351) water-insoluble components. Materials containing a high amount of water-insoluble components, with the exception of MH4-8, consisted of 45.4% (P139) to 68.6% (SH4-8) KOH-soluble components. KOH-soluble content in the other materials, with the exception of S176, ranged from 29.5% (P4-39) to 38.8% (MH4-8). S139, S176, SH4-8, P176, P351, and P4-39 exhibited a KOH-insoluble fraction (i.e. KOH-extracted cell walls) < 30%, with SH4-8 containing a percentage as low as 12.6%. The remaining materials contained > 30% KOH-insoluble components, with MH4-8 displaying a content as high as 45.0% (Fig. 3, Supplementary Table 1). Linear regression showed a strong positive relationship between the amount of water-insoluble cell wall components and *σ* (R² = 0.63) and a weak positive relationship with *ε* (R² = 0.24) (Fig. 4). A strong positive relationship was also found between the KOH-soluble material fraction and *σ* (R² = 0.64). Amounts of water-insoluble material components only displayed a weak relationship with *ε* (R² = 0.24), while the relationship between KOH-soluble components and *ε* was strong (R² = 0.66). Virtually no relationship could be found between KOH-insoluble material content and *σ* (R² = 0.07). By contrast, the same fraction displayed a moderate inverse relationship with *ε* (R² = 0.49). Together, a high amount of water-insoluble mycelium components and KOH-soluble cell wall components are good predictors of high strength and ductility.

**Fig. 5:**
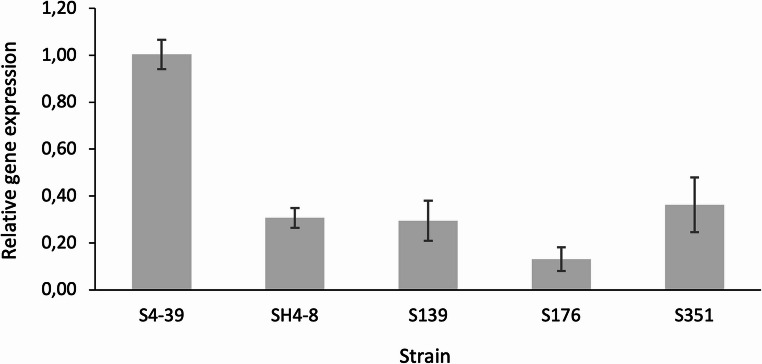
Expression of *sc3*, as determined by qPCR, in 3-day-old SCMM cultures of the dikaryotic strains as well as the monokaryotic strain 4–39. RNA of *arf1* was used as the reference gene and expression in 4–39 was set as 1

Previously, it was shown that deletion of the *sc3* hydrophobin gene in the *S. commune* 4–39 monokaryon increases *E* and *σ* 3–4 fold as a result of increased *ρ* of the mycelium material (Appels et al. 2018). Therefore, we quantified *sc3* expression to see whether the increased strength of the dikaryon mycelium materials is due to a higher *ρ* when compared to the 4–39 material because of reduced expression of the hydrophobin gene. Indeed, expression of *sc3* was between 2.8- and 7.7-fold lower in the mycelium of 3-day-old liquid shaken SCMM cultures of the dikaryons when compared to 4–39 (Fig. 5). These data strongly indicate that the increased strength of the dikaryotic mycelium materials is due to, at least in part, reduced Sc3 production when compared to the monokaryon.

## Discussion

Pure mycelium materials can be produced by drying a mycelium pulp obtained from liquid shaken cultures. Monokaryons of *S. commune* were so far preferred to produce such materials because of their high biomass production and their uniform materials due to the absence of (early stages of) fruiting bodies under any growth conditions. The presence of a second nucleus in dikaryotic hyphal compartments, however, enhances phenotypic stability (Clark and Anderson 2004; Kües 2000), masking recessive mutations that negatively impact biomass production and material properties. We here compared properties of pure mycelium materials from four dikaryotic strains of *S. commune* with those of the 4–39 monokaryon that is commonly used in mycelium material production (Appels et al. 2018;2020; d’Errico et al. 2024; 2025ab; van den Brandhof et al. 2024). Strain and medium composition determined biomass productivity and material properties. For instance, sheets displayed a *σ* of up to 47 MPa.

Results obtained in three media show that dikaryons can produce a biomass similar to that of a monokaryon. Monokaryon 4–39 was in the group of strains producing most biomass. High biomass of this strain was obtained in SCMM and PM. Dikaryon 176 produced a similar biomass as 4–39 in SCMM, while strains 139, 351, and H4-8 did so in PM. Lowest biomass was generally obtained in MM-N medium. This is probably explained by the presence of ammonium instead of asparagine without the addition of extra phosphate buffer to maintain the pH of the medium around 5.

The mycelial sheets of 4–39 grown in SCMM had a *σ* of 7.77 MPa, an *ε* of 1.89%, and an E of 0.61 GPa. Similar results were obtained when using MM-N and PM, showing that medium composition in this case had no effect on material properties of 4–39. In general, the medium had a low effect on mechanical properties of the different strains. Growth of strain 139 and H4-8 on PM resulted in a lower *σ* when compared to SCMM and SCMM and MM-N, respectively, while growth of strain 351 in PM resulted in a higher *σ* when compared to SCMM. *E* was only lower in the case of H4-8 on PM when compared to MM-N, while *ε* was lower on PM and MM-N when compared to SCMM in the case of strains 139 and H4-8. Data suggest that PM has the lowest performance, especially compared to SCMM. This may be due to the nitrogen source (asparagine in SCMM versus ammonium in MM-N and PM) and phosphate content. More detailed future studies should assess whether the nitrogen source and phosphate indeed impact the mechanical properties of pure mycelium materials.

Properties of the materials resulting from drying the mycelium of liquid shaken cultures of the *S. commune* 4–39 monokaryon were not very different from that of dikaryons of *Ganoderma resinaceum* and *Trametes betulina* (van den Brandhof et al. 2025). By contrast, we here showed that the *S. commune* dikaryons outperformed the monokaryon’s mechanical properties, producing 2.5- to 10-fold stronger (*σ*), up to 5.4-fold stiffer materials (*E*), and, in some cases, up to 5-fold more ductile materials (*ε*). The materials of strain 139 grown in SCMM and strain 351 grown in PM were the most performative, with *σ* values of 47.29 MPa and 47.16 MPa, *ε* values of 6.51% and 4.78% and *E* values of 1.73 GPa and 1.67 GPa, respectively. The strongest reported untreated pure mycelium material so far (*σ* = 40.4 MPa) was obtained by growing the *S. commune sc3* deletion mutant in a liquid static medium in the presence of light and high CO_2_ (Appels et al. 2018). Therefore, the materials obtained from strain 139 and 351 grown in SCMM and PM, respectively, are the strongest untreated mycelium materials produced so far. Future studies should reveal whether these differences are also found after chemical (d’Errico et al. 2024) or physical treatment (Sinha et al. 2025). In the latter case, materials were obtained with a *σ* of 67.6 MPa. This was achieved by passing mycelium through a three-roll mill. First, 1400 and 700 μm gaps were used between the rolls, followed by a second pass through 400 and 200 μm gaps. The resulting homogenous dispersion was dried into a film at high RH to enable regrowth during the drying process.

Our results indicate that the higher strength of the mycelium materials of the dikaryons is due to, at least partly, a high percentage of KOH-soluble cell wall components as well as a low *sc3* expression. The *sc3* gene is highly expressed during formation of aerial hyphae both in monokaryons and dikaryons of *S. commune* but expression is much lower when fruiting bodies are formed (Wösten and Wessels 2006). Deletion of the *sc3* gene in a *S. commune* monokaryon increases *σ* as a result of increased *ρ* of the mycelium material (Appels et al. 2018). This increased *ρ* is the result of collapse of air voids in the materials that are normally stabilized by the SC3 hydrophobin in the wild-type. The lower expression of *sc3* in the dikaryons used in our study would therefore also result in collapse of air voids and thereby increase *ρ* compared to the monokaryon, in turn, causing the higher *σ*. Reduced expression of *sc3* may also reduce the surface hydrophobicity of the mycelium material caused by self-assembly of this hydrophobin at the mycelium-air interface when the mycelium is dried into a sheet (Wösten et al., 1993).

The higher strength of the dikaryon materials may also be caused by the cell wall composition of the dikaryotic mycelium compared to that of the 4–39 monokaryon. Water-soluble mycelium material, mainly consisting of β-(1,3)(1,6)‐glucan (i.e. schizophyllan), protein, and cytoplasmic material is being removed by washing the cell walls with water (d’Errico et al. 2024; Ehren et al. 2020; Wessels et al. 1972). The percentage of water-insoluble material in the mycelium, corresponding to the water-extracted cell walls and consisting of both KOH-soluble and KOH-insoluble components (Safeer et al. 2023; Kleijburg et al. 2023), was a strong positive predictor of *σ*. In fact, a strong positive relationship was also found between the amount of KOH-soluble cell wall components and *σ*. This, together with the absence of a relationship between the KOH-insoluble fraction and *σ*, suggests a major role of KOH-soluble cell wall components in determining material strength. To illustrate this, the 4–39 material produced from PM-grown mycelium had a low *σ* (4.62 MPa) and 29.5% KOH-soluble material, while the material of 139 and 351 resulting from mycelium grown in SCMM and PM, respectively, had a *σ* of 47.29 and 47.16 MPa and 59.3% and 57.8% KOH-soluble cell wall content. How could the KOH-soluble material determine material strength? The KOH-soluble cell wall material mainly consists of the cell wall proteins (d’Errico et al., 2025a), an unknown polysaccharide X, and mannan, as well as part of the β‐(1,3)(1,6)‐glucan and α-(1–3)-glucan (Ehren et al., 2020). The KOH-insoluble material contains the other part of the latter two molecules, which may be in a different conformation compared to their KOH-soluble counterparts, as well as β‐(1,3)‐glucan and chitin (Ehren et al., 2020). The molecules in the KOH-soluble fraction, at least part of them, could have a higher propensity to form inter-molecular hydrogen bonds than the molecules found in the KOH-insoluble cell wall fraction and could thereby more effectively bind cell walls within the mycelium material together.

Interestingly, the KOH-soluble fraction also displays the strongest positive relationship with *ε* among all material components. By contrast, large amounts of KOH-insoluble cell wall components, consisting of chitin, α- and β-(1,3)-glucans, β-(1,3)-(1,6)-glucan, and fucan (Ehren et al. 2020) have a negative impact on *ε* and have no impact on *σ*. It has been shown that alkali-extracted chitin-glucan complexes that are present in the rigid backbone of the fungal cell wall yield materials with high tensile strength (Nawawi et al. 2019). The present data suggest that a large percentage of these molecules in untreated mycelium materials confers rigidity rather than strength, rendering the materials fragile. It is worth noting that material strength may not solely stem from the individual material components but could also arise from the architectural cell wall structure. Differences in this may, for instance, result in increased *ρ* (Appels et al. 2018).

Together, we here showed that *S. commune* dikaryons offer a phenotypically stable platform that yield stronger, stiffer, and more ductile materials compared to monokaryons. The properties of the dikaryon materials are similar to polymers, while the monokaryon 4–39 sheets are classified as a natural material when comparing the density and the stiffness of the material. These differences in material properties will favour either the monokaryon or the dikaryons for specific applications. Future studies should assess whether it is possible to obtain synergistic effects in changing the material’s strength and ductility by making combinations of physical and chemical treatments as well as genetic modification to reduce for instance *sc3* expression and/or increase the amount of KOH insoluble material in the cell wall.

## Supplementary Information

Below is the link to the electronic supplementary material.

Supplementary File 1 (DOCX 70.6 KB)

## Data Availability

Data will be made available by the authors upon request.

## References

[CR1] Antinori ME, Ceseracciu L, Mancini G, Heredia-Guerrero JA, Athanassiou A (2020) Fine-tuning of physicochemical properties and growth dynamics of mycelium-based materials. ACS Appl Bio Mater 9:1044–1051. 10.1021/acsabm.9b01031 10.1021/acsabm.9b01031 35019306

[CR2] Appels FVW, Dijksterhuis J, Lukasiewicz CE, Jansen KMB, Wösten HAB, Krijgsheld P (2018) Hydrophobin gene deletion and environmental growth conditions impact mechanical properties of mycelium by affecting the density of the material. Sci Rep 8:4703. 10.1038/s41598-018-23171-2 29549308 10.1038/s41598-018-23171-2 PMC5856774

[CR3] Appels FVW, van den Brandhof JG, Dijksterhuis J, de Kort GW, Wösten HAB (2020) Fungal mycelium classified in different material families based on glycerol treatment. Commun Biol 3:334. 10.1038/s42003-020-1064-4 32591629 10.1038/s42003-020-1064-4 PMC7320155

[CR4] Ashby MF (2005) Materials selection in mechanical design (3rd ed). Elsevier Butterworth-Heinemann, Burlington, MA.

[CR5] ASTM International. (2017). Standard Test Method for Young’s Modulus, Tangent Modulus, and Chord Modulus (ASTM E111-17). *ASTM International*. https://www.astm.org/

[CR6] Clark TA, Anderson JB (2004) Dikaryons of the basidiomycete fungus *Schizophyllum commune*: evolution in long-term culture. Genetics 167:1663–1675. 10.1534/genetics.104.027235 15342506 10.1534/genetics.104.027235 PMC1470993

[CR7] d’Errico A, Schröpfer M, Mondschein A, Wösten HAB (2025a). Characterization of the surface charge and reactivity of Schizophyllum commune mycelium material. Colloids Surf B Biointerfaces. 2025 Jun 4;254:114852. doi: 10.1016/j.colsurfb.2025.114852. 10.1016/j.colsurfb.2025.114852 40482386

[CR8] d’Errico A, Vonk PJ, Wösten HAB, Lugones LG (2025c) Transposition of a non-autonomous element into the Gβ gene of *Schizophyllum commune* causes the streak mutation. Fungal Genet Biol 179:104007. 10.1016/j.fgb.2025.104007 40447071 10.1016/j.fgb.2025.104007

[CR9] d’Errico A, Schröpfer M, Mondschein A, Safeer AA, Baldus M, Wösten HAB (2024) Cross-linking impacts the physical properties of mycelium leather alternatives by targeting hydroxyl groups of polysaccharides and amino groups of proteins. Heliyon 10:e36263. 10.1016/j.heliyon.2024.e36263 39253274 10.1016/j.heliyon.2024.e36263 PMC11382184

[CR10] d’Errico A, Land CA, Wissing M, Richrath RB, Wösten HAB (2025b) Polyglyceryl-4 caprate and polyglycerol-3 provide low mobility and enhance ductility in *Schizophyllum commune* mycelium materials. Colloids Surf. A Physicochem. Eng. Asp. 719:137055. 10.1016/j.colsurfa.2025.137055.

[CR11] Dons JJM, De Vries OMH, Wessels JGH (1979) Characterization of the genome of the basidiomycete *Schizophyllum commune*. Biochim Biophys Acta, Nucleic Acids Protein Synth 563:100–112. 10.1016/0005-2787(79)90011-X 10.1016/0005-2787(79)90011-x 574021

[CR12] Ehren HL, Appels FVW, Houben K, Renault MAM, Wösten HAB, Baldus M (2020) Characterization of the cell wall of a mushroom forming fungus at atomic resolution using solid-state NMR spectroscopy. The Cell Surface 6:100046. 10.1016/j.tcsw.2020.100046 33204900 10.1016/j.tcsw.2020.100046 PMC7649524

[CR13] Haneef M, Ceseracciu L, Canale C, Bayer IS, Heredia-Guerrero JA, Athanassiou A (2017) Advanced materials from fungal mycelium: fabrication and tuning of physical properties. Sci Rep. 10.1038/srep41292 28117421 10.1038/srep41292 PMC5259796

[CR14] Ivanova N (2022) Fungi for material futures: the role of design. In Deshmukh SK, Deshpande MV, Sridhar KR (eds) Fungal biopolymers and biocomposites: prospects and avenues. Springer Nature, Singapore, pp 209–251. 10.1007/978-981-19-1000-5_12

[CR15] Kleijburg FEL, Safeer AA, Baldus M, Wösten HAB (2023) Binding of micro-nutrients to the cell wall of the fungus *Schizophyllum commune*. The Cell Surface 10:100108. 10.1016/j.tcsw.2023.100108 38156043 10.1016/j.tcsw.2023.100108 PMC10753380

[CR16] Kleijburg FEL, Wösten HAB (2025) The versatility of *Schizophyllum commune* in nature and application. Fungal Biol Rev 53:100431. 10.1016/j.fbr.2025.100431

[CR17] Kleijburg FEL (2025) Functions of cell wall components of *Schizophyllum commune.* PhD Thesis, Utrecht University.

[CR18] Kües U (2000) Life history and developmental processes in the basidiomycete *Coprinus cinereus*. Microbiol Mol Biol Rev 64(2):316–353. 10.1128/mmbr.64.2.316-353.2000 10839819 10.1128/mmbr.64.2.316-353.2000 PMC98996

[CR19] Marian IM, Vonk PJ, Valdes ID, Barry K, Bostock B, Carver A, Daum C, Lerner H, Lipzen A, Park H, Schuller MBP, Tegelaar M, Tritt A, Schmutz J, Grimwood J, Lugones LG, Choi IG, Wösten HAB, Grigoriev IV, Ohm RA (2022) The transcription factor Roc1 is a key regulator of cellulose degradation in the wood-decaying mushroom *Schizophyllum commune*. mBio 13:e0062822. 10.1128/mbio.00628-22. 10.1128/mbio.00628-22 PMC9239231 35604096

[CR20] Nawawi WMFW, Lee KY, Kontturi E, Murphy RJ, Bismarck A (2019) Chitin nanopaper from mushroom extract: natural composite of nanofibers and glucan from a single biobased source. ACS Sustain Chem Eng 7:6492–6496. 10.1021/acssuschemeng.9b00721

[CR21] Ohm RA, de Jong JF, Lugones LG, Aerts A, Kothe E, Stajich JE, de Vries RP, Record E, Levasseur A, Baker SE, Bartholomew KA, Coutinho PM, Erdmann S, Fowler TJ, Gathman AC, Lombard V, Henrissat B, Knabe N, Kües U, Lilly WW, Lindquist E, Lucas S, Magnuson JK, Piumi F, Raudaskoski M, Salamov A, Schmutz J, Schwarze FW, vanKuyk PA, Horton JS, Grigoriev IV, Wösten HAB (2010a) Genome sequence of the model mushroom *Schizophyllum commune*. Nature Biotechnol 28:957–963. 10.1038/nbt.1643 10.1038/nbt.1643 20622885

[CR22] Ohm RA, de Jong JF, Berends E, Wang F, Wösten HAB, Lugones LG (2010b) An efficient gene deletion procedure for the mushroom-forming basidiomycete *Schizophyllum commune*. World J Microbiol Biotechnol 26:1919–1923. 10.1007/s11274-010-0356-0 20930926 10.1007/s11274-010-0356-0 PMC2940052

[CR23] Pfaffl MW (2001) A new mathematical model for relative quantification in real-time RT–PCR. Nucleic Acids Res 29:e45-e45. 10.1093/nar/29.9.e45 10.1093/nar/29.9.e45 PMC55695 11328886

[CR24] Raper JR, Miles PG (1958) The genetics of *Schizophyllum commune*. Genetics 43(3):530. 10.1093/genetics/43.3.530 17247776 10.1093/genetics/43.3.530 PMC1209900

[CR25] Safeer AA, Kleijburg F, Bahri S, Beriashvili D, Veldhuizen EJA, van Neer J, Tegelaar M, de Cock H, Wösten HAB, Baldus M (2023) Probing cell-surface interactions in fungal cell walls by high-resolution 1 H-detected solid-state NMR Spectroscopy. Chemistry 29:e202202616. 10.1002/chem.202202616 10.1002/chem.202202616 PMC10099940 36181715

[CR26] Schuurs TA, Dalstra HJ, Scheer JM, Wessels JGH (1998) Positioning of nuclei in the secondary mycelium of *Schizophyllum commune* in relation to differential gene expression. Fungal Genet Biol 23:150–161. 10.1006/fgbi.1997.1028 9578628 10.1006/fgbi.1997.1028

[CR27] Sinha A, Greca LG, Kummer N, Wobill C, Reyes C, Fischer P, Campioni S, Nyström G (2025) Living fiber dispersions from mycelium as a new sustainable platform for advanced materials. Adv Mater 37. 10.1002/adma.202418464 10.1002/adma.202418464 39998258

[CR28] Svensson SE, Ferreira JA, Hakkarainen M, Adolfsson KH, Zamani A (2021) Fungal textiles: Wet spinning of fungal microfibers to produce monofilament yarns. Sustain Mater Technol 28:e00256. 10.1016/j.susmat.2021.e00256

[CR29] Svensson SE, Abdollahi M, Moghadam FH, Kalita NK, Hakkarainen M, Wijayarathna EKB, Mohammadkhani G, Ferreira JA, Zamani A (2024) Valorization of bread waste to fungal-based products for medical textile and food applications. ACS Sustain Resour Managem 1:385–394. 10.1021/acssusresmgt.3c00021

[CR30] van Wetter MA., Wösten HAB, Sietsma JH, Wessels JG (2000) Hydrophobin gene expression affects hyphal wall composition in *Schizophyllum commune*. Fungal Genet Biol 31:99–104 (10.1006/fgbi.2000.1231). 10.1006/fgbi.2000.1231 11170739

[CR31] van den Brandhof JG, Wösten HAB, Tegelaar M (2024) Modulation of flexibility and shape recovery of living *Schizophyllum commune* pure mycelium material by hydration. Mater Today Commun 40:109784. 10.1016/j.mtcomm.2024.109784

[CR32] van den Brandhof JG, Hansen N, Hou C, Broers SC, Tegelaat M, Wösten HAB (2025) Characterization of pure mycelium materials from different mushroom-forming fungi. Antonie van Leeuwenhoek 118:121. 10.1007/s10482-025-02133-5 40699437 10.1007/s10482-025-02133-5 PMC12287172

[CR33] Vonk P, Escobar N, Wösten HAB, Lugones LG, Ohm RA (2019). High-throughput targeted gene deletion in the model mushroom *Schizophyllum commune* using pre-assembled Cas9 ribonucleoproteins. Sc Rep 9:7632. 10.1038/s41598-019-44133-2 10.1038/s41598-019-44133-2 PMC6529522 31113995

[CR34] Wessels JGH, Kreger DR, Marchant R, Regensburg BA, de Vries OMH (1972) Chemical and morphological characterization of the hyphal wall surface of the basidiomycete *Schizophyllum commune*. Biochimica et Biophysica Acta (BBA) 273:346–358. 10.1016/0304-4165(72)90226-7 5080323 10.1016/0304-4165(72)90226-7

[CR35] Wijayarathna ERKB, Mohammadkhani G, Soufiani AM, Adolfsson KH, Ferreira JA, Hakkarainen M, Berglund L, Heinmaa I, Root A, Zamani A (2022) Fungal textile alternatives from bread waste with leather-like properties. Resour Conserv Recycl 179:106041. 10.1016/j.resconrec.2021.106041

[CR36] Wösten HAB, De Vries OMH, Wessels JGH (1993) Interfacial self-Assembly of a fungal hydrophobin into a hydrophobic rodlet layer. Plant Cell 5:1567–1574. 10.1105/tpc.5.11.1567 12271047 10.1105/tpc.5.11.1567 PMC160386

[CR37] Wösten HAB, Wessels JGH (2006) The emergence of fruiting bodies in basidiomycetes. In: Kües U, Fischer R (eds) Growth, Differentiation and Sexuality. Springer, Berlin, pp 393–414

