# Supplementary material for: Strongest untreated mycelium materials produced by Schizophyllum commune dikaryons
Source: World J Microbiol Biotechnol. 2025 Oct 23;41(10):403. doi: 10.1007/s11274-025-04582-6 (PMC12549730; doi:10.1007/s11274-025-04582-6)
Supplement: Supplementary file 1 — (DOCX 70.6 KB) [file 11274_2025_4582_MOESM1_ESM.docx]

**Supplementary Information**

**Strongest untreated mycelium materials are produced by *Schizophyllum commune* dikaryons**

Antonio d’Errico, Jeroen G. van den Brandhof, Anna Bogomolova, Han A. B. Wösten

**Supplementary Table 1.** Different fractions of the materials expressed as a percentage of initial weight. Values are presented as mean ± SEM. Superscript letters indicate statistically significant differences with samples with different letters in the same column (p ≤ 0.05).

| **Condition** | **Water-soluble (%)** | **Water-resistant (%)** | **KOH-soluble (%)** | **KOH-resistant, %** |
| --- | --- | --- | --- | --- |
| **S4-39 (a)** | 36.3 ± 1.5^b,e,g,i,j,l,n^ | 63.7 ± 1.5^b,e,g,i,j,l,n^ | 30.8 ± 1.0^b,c,e,g,l,n^ | 32.9 ± 2.36^b,e,i,j,m,n^ |
| **S139 (b)** | 14.7 ± 1.7^a,c,d,f,h,i,k-m,o^ | 85.3 ± 1.7^a,c,d,f,h,i,k-m,o^ | 59.2 ± 1.5^a,c-f,h-m,o^ | 26.07 ± 3.27^a,d-j^ |
| **S176 (c)** | 30.4 ± 3.5^b,e,g,j,k,m,n^ | 70.0 ± 3.5^b,e,g,j,k,m,n^ | 40.0 ± 3.0^a,b,e,f,g,k,n^ | 29.62 ± 1.35^e,f,i,j^ |
| **S351 (d)** | 33.9 ± 0.3^b,e,g,j,k,l,n^ | 66.1 ± 0.3^b,e,g,j,k,l,n^ | 32.0 ± 0.6^b,e,g,l,n^ | 34.08 ± 1.34^b,e,j,m,n^ |
| **SH4-8 (e)** | 18.8 ± 1.1^a,c,d,f,h,I,k,m,o^ | 81.2 ± 1.1^a,c,d,f,h,I,k,m,o^ | 68.6 ± 1.4^a-d,f-o^ | 12.6 ± 1.44^a-d,f-o^ |
| **M4-39 (f)** | 30.2 ± 0.9^b,e,g,j,k,m,n^ | 69.8 ± 0.9^b,e,g,j,k,m,n^ | 31.3 ± 0.4^b,c,e,g,l,n^ | 38.6 ± 1.2^b,c,e,j-o^ |
| **M139 (g)** | 11.5 ± 1.0^a,c,d,f,h,i,k-m,o^ | 88.5 ± 1.0^a,c,d,f,h,i,k-m,o^ | 53.1 ± 3.2^a,c-f,h-k,m,o^ | 35.4 ± 2.2^b,e,j,k,m,n^ |
| **M176 (h)** | 34.4 ± 0.4^b,e,g,j,l,n^ | 65.6 ± 0.4^b,e,g,j,l,n^ | 33.0 ± 0.4^b,e,g,l,n^ | 32.6 ± 0.1^b,e,I,j,m,n^ |
| **M351 (i)** | 27.4 ± 1.1^a,b,e,g,j,k,m,n^ | 72.6 ± 1.1^a,b,e,g,j,k,m,n^ | 32.5 ± 0.8^b,e,g,l,n^ | 40.1 ± 0.3^a-c,e,h,k-o^ |
| **MH4-8 (j)** | 16.2 ± 0.9^a,c,d,f,h,i,k,m,o^ | 83.8 ± 0.9^a,c,d,f,h,i,k,m,o^ | 38.8 ± 0.4^b,e,g,k,n^ | 45.0 ± 0.6^a-h,k-o^ |
| **P4-39 (k)** | 41.8 ± 1.2^b-g,I,j,l,n,o^ | 58.2 ± 1.2^b-g,I,j,l,n,o^ | 29.5 ± 1.3^b,c,e,g,j,l,n^ | 28.7 ± 0.7^e-g,i,j^ |
| **P139 (l)** | 23.0 ± 1.2^a,b,d,g,h,k,m,o^ | 77.0 ± 1.2^a,b,d,g,h,k,m,o^ | 45.4 ± 2.3^a,b,d-f,h,I,k,m-o^ | 31.6 ± 1.5^e,f,i,j^ |
| **P176 (m)** | 39.1 ± 0.8^b,c,e-g,i,j,l,n^ | 60.9 ± 0.8^b,c,e-g,i,j,l,n^ | 34.7 ± 0.8^b,e,g,l,n^ | 26.2 ± 0.5^a,d-j^ |
| **P351 (n)** | 16.1 ± 1.1^a,c,d,f,h,i,k,m,o^ | 83.9 ± 1.1^a,c,d,f,h,i,k,m,o^ | 57.8 ± 1.8^a,c,d-f,h-m,o^ | 26.1 ± 1.6^a,d-j^ |
| **PH4-8 (o)** | 33.6 ± 1.5^b,e,g,j,k,l,n^ | 66.4 ± 1.5^b,e,g,j,k,l,n^ | 36.4 ± 1.0^b,e,g,l,n^ | 30.0 ± 0.8^e,f,i,j^ |


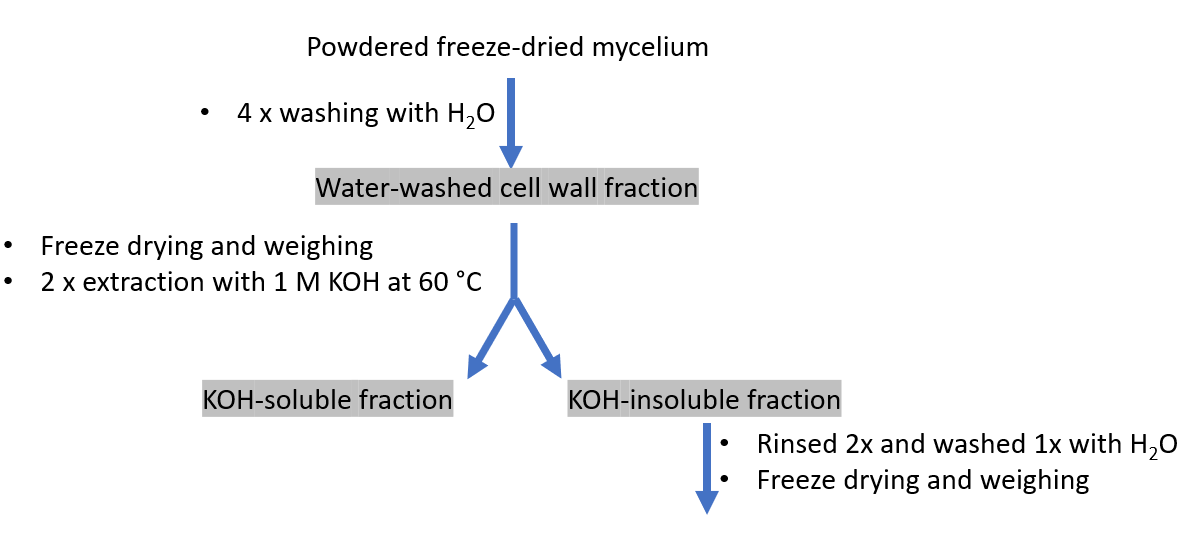


**Supplementary Figure 1.** Flowchart showing the preparation of water-washed and KOH-extracted cell walls.
